# Bacterial profiling of White Plague Disease in a comparative coral species framework

**DOI:** 10.1038/ismej.2013.127

**Published:** 2013-08-08

**Authors:** Cornelia Roder, Chatchanit Arif, Till Bayer, Manuel Aranda, Camille Daniels, Ahmed Shibl, Suchana Chavanich, Christian R Voolstra

**Affiliations:** 1 Red Sea Research Center, King Abdullah University of Science and Technology, Thuwal, Saudi Arabia; 2 Department of Marine Science, Faculty of Science, Chulalongkorn University, Reef Biology Research Group, Bangkok, Thailand

**Keywords:** 16S rRNA gene microarray, Gulf of Thailand, *Pavona duerdeni*, *Porites lutea*, coral disease, White Plague Disease (WPD)

## Abstract

Coral reefs are threatened throughout the world. A major factor contributing to their decline is outbreaks and propagation of coral diseases. Due to the complexity of coral-associated microbe communities, little is understood in terms of disease agents, hosts and vectors. It is known that compromised health in corals is correlated with shifts in bacterial assemblages colonizing coral mucus and tissue. However, general disease patterns remain, to a large extent, ambiguous as comparative studies over species, regions, or diseases are scarce. Here, we compare bacterial assemblages of samples from healthy (HH) colonies and such displaying signs of White Plague Disease (WPD) of two different coral species (*Pavona duerdeni* and *Porites lutea*) from the same reef in Koh Tao, Thailand, using 16S rRNA gene microarrays. In line with other studies, we found an increase of bacterial diversity in diseased (DD) corals, and a higher abundance of taxa from the families that include known coral pathogens (Alteromonadaceae, Rhodobacteraceae, Vibrionaceae). In our comparative framework analysis, we found differences in microbial assemblages between coral species and coral health states. Notably, patterns of bacterial community structures from HH and DD corals were maintained over species boundaries. Moreover, microbes that differentiated the two coral species did not overlap with microbes that were indicative of HH and DD corals. This suggests that while corals harbor distinct species-specific microbial assemblages, disease-specific bacterial abundance patterns exist that are maintained over coral species boundaries.

## Introduction

One of the most recognized features of tropical, shallow-water corals is their symbiosis with photosynthetic unicellular algae (zooxanthellae) that provide photosynthetically fixed carbon to satisfy their host's respiratory requirements (Muscatine and Cernichiari 1969) and facilitate calcification (Gattuso *et al.*, 1999). Corals also live in association with numerous other microorganisms such as bacteria, archaea, protists, endolithic algae, fungi and viruses (Rosenberg *et al.*, 2007), the significance of which is only partially understood (Bourne *et al.*, 2009; Kimes *et al.*, 2010). The sum of all organisms is referred to as the coral holobiont (Rosenberg *et al.*, 2007).

It is now being recognized that bacteria contribute significantly to the biology of higher-order organisms (Ezenwa *et al.*, 2012), and accordingly, bacteria associated with corals are considered a vital component of the coral holobiont. Their potential roles include nitrogen fixation (Lesser *et al.*, 2004), decomposition of organic materials (DiSalvo, 1969), production of antibiotic compounds (Kelman *et al.*, 2006; Ritchie, 2006) and occupation of space to prevent colonization by pathogens (Ritchie and Smith, 2004). Coral-associated bacteria have been shown to be host species-specific, diverse and complex (Rohwer *et al.*, 2001, 2002; Sunagawa *et al.*, 2010), and this assemblage comprises a unique signature that differs from bacterial communities in the surrounding water column (Rohwer *et al.*, 2001; Frias-Lopez *et al.*, 2002; Bourne and Munn, 2005).

Several studies have been conducted that highlight the role of bacteria in coral diseases (Denner, 2003; Barash *et al.*, 2005; Rosenberg *et al.*, 2007; Bourne *et al.*, 2009; Meron *et al.*, 2009; Sunagawa *et al.*, 2009; Kimes *et al.*, 2010; Cardenas *et al.*, 2012; Cróquer *et al.*, 2013). Coral diseases appear as changes in tissue color in the form of patches or bands on the coral surface, associated with subsequent tissue damage, necrosis and tissue loss (Richardson, 1998). In many areas, disease outbreaks have led to massive die-offs of reef-building corals that resulted in habitat loss for reef-associated organisms, with propensity for irreversible ecosystem change (Richardson, 1998; Richardson *et al.*, 2001; Pandolfi *et al.*, 2005; Weil *et al.*, 2006). To date, the exact number of coral diseases remains unknown (Pollock *et al.*, 2011). Their characterization is mainly based on field observations of altered phenotypes. As a result, the same disease might have been defined several times and in different ways depending on the species or the region affected (Richardson, 1998). For most diseases, our knowledge on causative agents, modes of transmission or disease reservoirs is missing (Weil *et al.*, 2006). It is unknown whether the same pathogens cause similar/same disease characteristics in different coral hosts or whether the same shifts in microbial assemblages result in the same disease phenotype in different coral species (Lesser *et al.*, 2007; Rosenberg and Kushmaro, 2011). Furthermore, it is not known whether diseases with a similar phenotype are caused by similar underlying mechanisms, that is, if they are associated with comparable bacterial changes or species (Lesser *et al.*, 2007). Answers to these questions might not only enable a clearer disease nomenclature but will also result in a better understanding of the mechanisms driving coral disease outbreak and progression and will eventually lead to a better understanding of coral holobiont pathology (Rogers, 2010; Pollock *et al.*, 2011).

White Plague Disease (WPD) is one of the first described coral diseases (Dustan, 1977). Records show that WPD was responsible for several virulent outbreaks, and it is held responsible for major reef declines worldwide, especially in the Caribbean (Richardson *et al.*, 1998b; Aronson and Precht, 2001; Richardson *et al.*, 2001; Navas-Camacho *et al.*, 2010; Pollock *et al.*, 2011). Corals affected by a WPD phenotype show a pronounced line of bright, white tissue that separates the colored (living) part of the coral from bare, rapidly algal-colonized skeleton (Richardson *et al.*, 2001). Three types of WPD, I (Dustan, 1977), II (Richardson *et al.*, 1998b) and III (Richardson *et al.*, 2001), have been described that differ in the rate of progression across a coral's surface and affect different species (Richardson *et al.*, 2001; Sutherland *et al.*, 2004). Richardson *et al.* (1998a) initially suggested a species of *Sphingomonas* as the causative pathogen but Denner (2003) proposed *Aurantimonas coralicida* as the final WPD-causing pathogen in corals from the Caribbean. Similarly, *Thalassomonas loyana* (Thompson *et al.*, 2006) has been proposed to be the causative agent of White Plague-like disease in the Red Sea. However, neither of these bacteria could be unequivocally verified as the responsible pathogen in subsequent studies (Pantos *et al.*, 2003; Barash *et al.*, 2005; Sunagawa *et al.*, 2009; Cardenas *et al.*, 2012). Consequently, it is debatable whether a definitive pathogen for WPD exists or whether different pathogens or bacterial consortia produce a similar disease phenotype in different coral species. Given the inherent difficulties of assigning a pathogen to WPD, and thereby proving a causal relationship, Willis *et al.* (2004) suggested that coral diseases from the Great Barrier Reef (and by extension the Indo-Pacific) that produce a phenotype of white bands of tissue and/or skeleton should be referenced collectively as White Syndrome, unless the underlying disease etiology is known. Here we employed an alternative approach and tested whether healthy (HH) and diseased (DD) coral colonies displaying a WPD-characteristic phenotype (Dustan, 1977; Richardson *et al.*, 2001) from the Indo-Pacific share similarities in underlying microbial community patterns and are comparable to WPD-affected corals and studies from the Caribbean.

Sunagawa *et al.* (2009) was the first study that used 16S rRNA gene microarrays (PhyloChips, Second Genome) to assess bacterial community changes in WPD in *Montastraea faveolata* and demonstrated the overall feasibility of the method. In this study, we used PhyloChips to profile microbial communities of HH and DD colonies of two coral species (*Porites lutea* and *Pavona duerdeni*) displaying signs of WPD collected from the same reef in Koh Tao, Thailand. Our aim was to examine microbial community differences within and between species and between coral health states (HH vs DD). Additionally, 16S rRNA gene clone library sequencing was conducted to compare the two different methods for assaying coral-associated bacterial community structure.

## Materials and methods

### Sample collection

Sampling took place offshore of Sairee Beach (10.097908′ N, 99.825163′ E), Koh Tao Island, in the Gulf of Thailand during non-monsoon season in January 2011. Tissue was sampled from three HH colonies and three colonies displaying signs of WPD between 4 and 7 m depth by SCUBA (Cressi, Genoa, Italy) using hammer and chisel from the two coral species *P. duerdeni* and *P. lutea*. DD colonies displayed an abrupt band of white, exposed coral skeleton that separated living tissue from algal-colonized dead coral skeleton. Samples from HH colonies were chiseled off the uppermost part of the colonies, while samples displaying WPD signs were taken from the interface of HH and DD tissue. All samples were handled wearing gloves and directly transferred into sterile Whirl-Pak (Nasco, Fort Atkinson, WI, USA) sampling bags. On board, corals were rinsed with filtered seawater (0.22 μm) and wrapped in aluminum foil. One liter of seawater was sampled from the water column above the reef and filtered (20 mm Hg) onto 0.22 μm Durapore PVDF filters (Millipore, Billerica, MA, USA). All samples were immediately flash frozen in liquid nitrogen on board and stored at −80 °C until subsequent DNA extraction.

### DNA extraction

Coral samples were crushed to powder in liquid nitrogen using autoclaved mortars and pestles. Aliquots of 50–100 mg of coral powder and the disrupted filter holding the microbial community of the water column were utilized for DNA extraction using the DNeasy Plant Mini Kit (Qiagen, Hilden, Germany). DNA concentrations were quantified on a NanoDrop 2000C spectrophotometer (Thermo Fisher Scientific, Waltham, MA, USA) and with a Qubit fluorometer using the Quant-IT dsDNA Broad Range Assay Kit (Invitrogen, Carlsbad, CA, USA).

### PhyloChip PCR and hybridization

DNAs were shipped on dry ice to Second Genome Inc. (San Bruno, CA, USA) for assaying on the PhyloChip G3 platform. Up to 500 ng of PCR product was applied to each PhyloChip G3 following previously described procedures (Hazen *et al.*, 2010). Briefly, the 16S rRNA amplicons and a mixture of amplicons at known concentrations (spike-mix) were combined, fragmented using DNAse1 (Invitrogen) and biotin-labeled using the recommended protocol for Affymetrix Prokaryotic Arrays (Santa Clara, CA, USA). Labeled products were hybridized overnight at 48 °C and 60 r.p.m. The arrays were washed, stained and scanned as previously described (Hazen *et al.*, 2010).

### PhyloChip data transformation and normalization

Details on probe selection, probe scoring, data acquisition and preliminary data analysis are according to Hazen *et al.* (2010). Array fluorescence intensities were collected as integer values ranging from 1 to 65 536 (2^0^–2^16^). Subsequent log2 transformation yielded decimal numbers ranging from 0 to 16 that were multiplied by 1000 yielding a range of 0–16 000 (HybScore). To correct for uneven hybridization, differences in hybridization intensities and scale, intensity HybScores were loess-normalized using the normalize.loess function in the affy package (Gautier *et al.*, 2004) in the R statistical environment (R Development Core Team, 2010). A microbial taxon was regarded present if it was identified in two of the three replicates of any species/condition combination (*P. duerdeni* HH, *P. duerdeni* DD, *P. lutea* HH, *P. lutea* DD) or determined present in the water sample based on the method in Hazen *et al.* (2010). Of the 59 222 bacterial operational taxonomic units (OTUs) assayed on the PhyloChip, 29 103 were present over all samples (Supplementary Tables S1 and S2).

### PhyloChip data analysis

To visualize similarities within and between species-condition combinations, a multidimensional scaling (MDS) plot based on Bray–Curtis distances of OTU abundance data was generated using the libraries MASS and vegan in the R statistical environment (R Development Core Team, 2010). A corresponding two-way crossed (species and condition) analysis of similarity (ANOSIM) on the basis of the same resemblance matrix (Bray–Curtis distances of OTU abundances between samples) and using 999 permutations was conducted in the PRIMER v6 (PRIMER-E Ltd, Ivybridge, UK) software (Clarke, 1993). The degree of correspondence between the distances among points implied by MDS was measured by a stress function of the form √ΣΣ(f(*x*_ij_)−*d*_ij_)^2^/scale. In the equation, *d*_ij_ refers to the Bray–Curtis distance between samples, f(*x*_ij_) is some function of the input data and scale refers to a constant scaling factor used to keep stress values between 0 and 1. The smaller the stress, the better the representation. Normalized HybScores were analyzed using the TM4 software (Dana-Farber Cancer Institute, Boston, MA, USA) (Saeed *et al.*, 2003). A two-way factorial analysis of variance (ANOVA) was conducted based on the 14 213 OTUs present in the coral samples to determine differentially abundant OTUs between HH and DD samples and between species, as well as combinations thereof. Corresponding *P*-values were false discovery rate adjusted via R software package QVALUE (Storey, 2002) with a false discovery cutoff rate of 10%. Hierarchical clustering using Euclidean distance was performed on HybScores averaged over triplicates, and a heatmap was generated using the heatmap.2 function in the gplots package in the statistical environment R (R Development Core Team, 2010). Bacterial family over-representation was analyzed via chi-square test with Yates' correction by comparing number of differentially abundant OTUs in a family in relation to all OTUs assayed for that family on the PhyloChip. Only families that were represented by at least five taxa were considered.

### Cloning and sequencing

16S rRNA genes PCRs were run using coral DNAs according to the PhyloChip PCR protocol and primers (Hazen *et al.*, 2010) to generate clone libraries. PCR products were cleaned with the MinElute PCR Purification Kit (Qiagen). Clones for each sample were produced with a PCR Cloning Kit (Qiagen) and picked into a 96-well plate, which contained a 25-μl mastermix that consisted of 1 × Multiplex Mix (Qiagen), 0.2 μM each of M13F (−43: 5′-AGGGTTTTCCCAGTCACGACGTT-3′) and M13R (−49: 5′-GAGCGGATAACAATTTCACACAGG-3′) primers and DNAse-RNAse-free water. M13 PCR conditions were 94 °C for 15 min, 30 cycles of 94 °C for 30 s, 55 °C for 90 s, 72 °C for 90 s, and a final extension of 72 °C for 10 min. The 16S rRNA clones were sequenced bi-directionally with M13F (−21: 5′-TGTAAAACGACGGCCAGT-3′) and M13R (−29: 5′-CAGGAAACAGCTATGACC-3′) on an ABI 3730xl (Applied Biosystems by Life Technologies, Foster City, CA, USA) at the KAUST BioScience Core Facility. Sequence data have been submitted to the GenBank database under accession numbers KC527063—KC527539.

### Clone library analysis

16S rRNA gene sequences represented on the PhyloChip microarray were extracted from the Greengenes 2011 sequence data set (McDonald *et al.*, 2012) resulting in 59 112 sequences, which were used to create a BLAST database. Clone sequences were quality-trimmed, assembled, aligned, and checked for orientation in Codon Code Aligner (Codon Code Corporation, Centerville, MA, USA) to obtain full-length 16S rRNA genes. 16S rRNA genes were queried with BLAST 2.2.26+ (BLASTN) (Altschul *et al.*, 1990) to assign a taxonomic level of classification to the clone sequence as described in DeSantis *et al.* (2007). Briefly, clone sequence and BLASTN hit were aligned to the Greengenes 16S rRNA genes alignment using NAST (DeSantis *et al.*, 2006), and a Lane mask (Lane, 1991) was applied using mothur (Schloss *et al.*, 2009). DNADIST (Felsenstein, 1989) was used to calculate the sequence similarity between sequence pairs using the F84 model assuming a transition/transversion ratio of 2.0 and an A, C, G, T base frequency of 0.2509, 0.2276, 0.3156, 0.2057, respectively. The obtained similarity values were split into taxonomic groups according to the DNADIST percent similarity (Phylum (⩾80%), Class (⩾85%), Order (⩾90%), Family (⩾92%), Subfamily (⩾94%), OTU (⩾97%)). In addition to the PhyloChip subset of 16S rRNA gene sequences, cloned 16S rRNA genes were also compared with the full Green genes 2011 database.

## Results

### PhyloChip and clone library comparison

To determine the amount of bacterial taxa that were not assayed on the PhyloChip, we conducted a comparison of PhyloChip to clone library sequencing (Table 1). On the phylum level, all sequences identified by clone libraries were also detected by the PhyloChip. Similarly, for all lower taxonomic ranks, the percentage of assigned 16S rRNA clones via Greengenes database and the PhyloChip was highly similar. It is worth noting that only about 50% of all 16S rRNA genes could be annotated on the family level and only about 40% of 16S rRNA genes on the OTU level, irrespective of the technique used. At the OTU level (⩾97% similarity), the PhyloChip missed only 15 clones that were successfully assigned to a 16S rRNA sequence via the Greengenes database.

### Patterns of bacterial richness and diversity in healthy and diseased corals

Of the 59 222 microbial OTUs assayed on the PhyloChip G3 microarray, 29 103 were present in our samples (Table 2). Of these, 14 213 were present in corals and 18 418 OTUs were found in reef water. DD fragments had about one-third more bacterial OTUs than their HH counterparts, and *P. lutea* contained more than double the amount of bacterial OTUs than *P. duerdeni* irrespective of the health state (that is, HH or DD).

**Figure 1:**
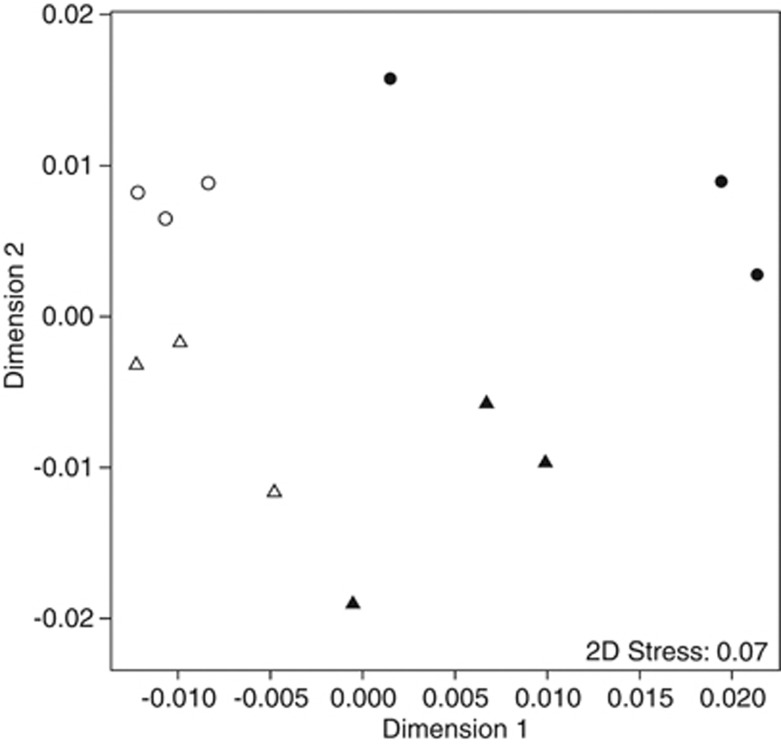
Multidimensional (MD) scaling plot based on Bray*–*Curtis distances of normalized PhyloChip HybScores of healthy (circles) and diseased (triangles) specimens of the corals *P. duerdeni* (white) and *P. lutea* (black) illustrating the similarity of associated bacterial communities. Stress represents the goodness of fit of the data onto the MD ordination.

To elucidate patterns of species and health state differences, we compared species-condition differences using ANOSIM (Table 3) and plotted the results in a MDS ordination (Figure 1). Samples significantly (*P*<0.01) clustered according to coral species and condition (Table 3) as visualized by a partitioning of the samples along the two axes in the MDS ordination (Figure 1), indicating that microbial communities in corals separate according to species and disease. However, we found varying distances between replicates of species and conditions that emphasize that a high degree of natural variation between coral colonies seems to exist. The strength of difference (*R*) between microbial communities of the two coral species (*P. duerdeni* vs *P. lutea*) and between the two health conditions (HH vs DD) was equally significant and displayed a similar and high *R* value (*R*=0.65 for species, *R*=0.54 for condition; Table 3). It is important to note that the difference between health states is irrespective of the coral species, and hence, a strong pattern of microbial community stratification in HH and DD coral tissue exists that is consistent over coral species boundaries.

### Differentially abundant OTUs between species and disease states

A two-way ANOVA between all four species-condition combinations (*P. duerdeni* HH, *P. duerdeni* DD, *P. lutea* HH, *P. lutea* DD) identified a total of 1003 OTUs that were differentially abundant between coral species and 629 OTUs that were differentially abundant between HH and DD samples (Table 3). The difference between coral species and conditions was similar, although species differences were more pronounced. This result corroborates the ANOSIM analysis. Notably, none of the OTUs identified was significant in both comparisons (that is, showed a species × condition interaction; Table 3, Supplementary Table S3), indicating that OTUs that are different between species are distinct from OTUs that are different between health states.

The majority of OTUs that showed significant differences in abundance between the two coral species were more abundant in *P. duerdeni* than in *P. lutea* (Supplementary Figure S1A). This was true for HH samples (655 vs 348 OTUs), as well as for DD samples (651 vs 352 OTUs). We aggregated the 1003 bacterial OTUs to the level of family. A chi-square analysis showed an over-representation of bacteria belonging to the families Bacillaceae, Comamonadaceae, Enterobacteriaceae, Lachnospiraceae and Streptococcaceae among the differentially abundant OTUs that separate coral species (df=2, all *P*<0.01, Table 4).

About two-thirds of OTUs significantly different between HH and DD were more abundant in DD specimens (*P. duerdeni*: 428 OTUs DD vs 201 OTUs HH; *P. lutea*: 429 OTUs DD vs 200 OTUs HH; Supplementary Figure S1B). Comparison of HH and DD samples via congregated family fold-change differences of the 629 OTUs showed a higher abundance of bacteria belonging to the families Comamonadaceae, Enterobacteriaceae and Streptococcaceae in HH samples (among others). In contrast, bacteria belonging to the families Colwelliaceae, Pseudomonadaceae, Rhizobiaceae and Rhodobacteraceae were over-represented and more abundant in DD samples (chi-square, df=2, all *P*<0.01, Table 5). Changes in abundance were highest for bacteria belonging to the families Oceanospirillaceae, Rhodobacteraceae and Vibrionaceae (all >4-fold more abundant in DD tissues for both coral species).

## Discussion

Coral-associated microbes constitute an essential component in coral holobiont functioning (Rosenberg *et al.*, 2007). In particular, bacteria seem to have important roles in coral health and disease that still need to be further defined. One approach to identify common bacterial species is to conduct microbial studies in a comparative coral species framework. By choosing two species from the same coral reef, we limited variation in environmental variables in order to focus on the difference between coral species and coral health states. Here we characterized the abundance patterns of bacterial OTUs associated with HH and DD samples of *P. duerdeni* and *P. lutea* in a standardized comparison via 16S rRNA gene microarrays. The general feasibility of the PhyloChip platform to assess microbial community patterns in coral disease has been established by Sunagawa *et al.* (2009). With regard to taxonomic diversity and identification of OTUs from corals collected at Sairee Beach in Thailand, PhyloChip microarrays yielded comparable results to clone library sequencing efforts. Both methods identified all OTUs to the phylum level and half of the OTUs to the family level, whereas about 60% of all the sequences failed to be annotated to the level of OTU with either method. We found a higher number of OTUs in our study (between 2756 OTUs in *P. duerdeni* HH and 10 848 OTUs in *P. lutea* DD) in comparison to sequence-based studies that looked at bacterial diversity in corals (for example, Barott *et al.* (2011): between 163 and 461 OTUs per sample; Cardenas *et al.* (2012): between 256 and 378 OTUs per sample; Koren and Rosenberg (2006): 400 OTUs; Lins-de-Barros *et al.* (2010): 354 OTUs). However, our estimates are well in line with estimates from Kellogg *et al.* (2012) that identified between 1112 and 9240 OTUs with PhyloChips in a comparison of sampling methods for coral microbial community analysis.

Our data suggest that a lower bacterial diversity and abundance is associated with HH corals, which has also been reported by Pantos *et al.* (2003), Sunagawa *et al.* (2009) and Cróquer *et al.* (2013). We identified Pseudomonadaceae and Rhodobacteraceae as prominent families promoted in colonies displaying WPD signs. Rhodobacteraceae have been proposed to be opportunistic due to uncontrolled propagation in disease by Sunagawa *et al.* (2009). Furthermore, bacterial taxa of the family Vibrionaceae were more abundant in DD samples as has been shown previously (Sunagawa *et al.*, 2009; Mouchka *et al.*, 2010; Pollock *et al.*, 2011). Cardenas *et al.* (2012) conducted a study with a similar experimental design and compared the microbiome of HH and WPD-affected corals from two species (*Diploria strigosa* and *Siderastrea siderea*) in the Caribbean via 16S rRNA gene amplicon sequencing, but the authors did not find consistent bacterial shifts over coral species. The use of pooled replicates by Cardenas *et al.* (2012) for the different conditions and species might have influenced the ability to statistically test for coral species or condition specificity. Alternatively, WPD-affected corals in the Caribbean might display a different pattern. We did not find *A. coralicida* (GenBank ID EF512716.1), the putative WPD pathogen from the Caribbean, in any of the coral samples using clone libraries or the PhyloChip microarray. Also, *T. loyana* (GenBank ID AY643537.2), a proposed causative agent of White Plague-like disease from the Red Sea, was neither identified during our cloning efforts, nor detected on the microarray. This is consistent with results of other WPD-investigating studies that failed to discover either of these bacteria (Pantos *et al.*, 2003; Sunagawa *et al.*, 2009; Cardenas *et al.*, 2012), which might be due to investigating phenotypically similar but not identical diseases (Willis *et al.*, 2004; Lesser *et al.*, 2007). It could also be argued that pathogens are subject to evolutionary change, which has been shown in other coral diseases (Reshef *et al.*, 2008). In this regard, the loss of pathogenicity due to changes in environmental conditions (Meron *et al.*, 2009), repression through a newly, more favorably structured holobiont microbial assemblage (Reshef *et al.*, 2006) or control through bacteriophages (Cohen *et al.*, 2013) could be possible explanations.

When comparing HH and DD samples, there is a clear trend from bacterial communities low in diversity and abundance (HH) to mixed and variable assemblages with high numbers of unclassified bacteria (DD), many of which were also identified in the surrounding water (data not shown). Most notably, we found no overlap between OTUs differentially abundant between coral species and their health states. Our data indicate that phenotypically similar coral diseases are accompanied by a common shift in bacterial communities in the two different coral species collected from the same reef. At the same time, corals display species-specific bacterial communities that are different from disease-associated bacteria. Health and disease were as strong a discriminator between samples as species. One important consequence is that microbial community patterns (‘bacterial footprints') might exist, which classify HH and DD coral specimens over species boundaries. In this regard, our study represents an approach to compare and analyze microbial assemblages of coral disease in a standardized framework (that is, via PhyloChip profiles) that might aid in the classification and categorization of coral diseases. Future studies should incorporate measures over geographical distances in the same and different species in order to understand whether these patterns are only regionally or globally conserved.

## Figures and Tables

**Table 1:** Number of distinct taxonomic ranks identified by PhyloChip in comparison to clone library sequencing of a pool of 96 clones from each sample (*n*=477)

*Taxonomic rank* *(% cutoff)*	*Clones classified in Greengenes database (2011)*		*Clones detected by PhyloChip*	
	*N*	*%*	*N*	*%*
Phylum (⩾80%)	474	99.37	475	99.58
Class (⩾85%)	266	55.77	267	55.97
Order (⩾90%)	254	53.25	254	53.25
Family (⩾92%)	249	52.20	248	51.99
Subfamily (⩾94%)	230	48.22	228	47.80
OTU (⩾97%)	200	41.93	185	38.78
unclassified	3	0.63	2	0.42

Abbreviation: OTU, operational taxonomic unit.

**Table 2:** Number of detected OTUs over all samples with PhyloChip microarrays

*PhyloChip*	*No. of OTUs*
Detected in coral and water	29 103
Detected in coral	14 213
in *Pavona duerdeni* HH	2756
in *Pavona duerdeni* DD	4434
in *Porites lutea* HH	7580
in *Porites lutea* DD	10 848
Detected in water	18 418

Abbreviations: DD, diseased; HH, healthy; OTU, operational taxonomic unit.

**Table 3:** Summary statistics of two-way crossed ANOSIM and two-way ANOVA

*ANOSIM (based on Bray–Curtis distances)*
Differences between species (*P. duerdeni* vs *P. lutea*)
Strength of difference R: 0.65
Significance *P*<0.01

Differences between conditions (HH vs DD)
Strength of difference R: 0.54
Significance *P*<0.01

*ANOVA (14 213 OTUs, FDR <0.1)*	*No. of OTUs*
Species significant (*P. duerdeni* vs *P. lutea*)	1003
Condition significant (HH vs DD)	629
Interaction significant (species × condition)	0

Abbreviations: ANOSIM, analysis of similarity; ANOVA, analysis of variance; DD, diseased; FDR, false discovery rate; HH, healthy.

**Table 4:** Over-/under-representation of bacterial families of OTUs differentially abundant between coral species, and congregated fold-change differences between healthy and diseased specimens of *P. duerdeni* and *P. lutea* (only families that were represented by at least five bacterial taxa were considered)

*Bacterial family*	*OTU count ANOVA (total 1003)*	*OTU count PhyloChip (total 14 213)*	*Chi-square*	P-*value*	*Mean fold-change difference between healthy corals*	*Mean fold-change difference between diseased corals*
*More abundant in P. duerdeni*						
Aquabacteriaceae	12	310	3.9220	<0.05	1.92	1.87
Bacillaceae	38	264	16.9856	<0.0001	2.04	1.69
Bacteroidaceae	5	37	1.1628	ns	1.23	1.13
Burkholderiaceae	7	146	0.6845	ns	1.45	1.82
Clostridiaceae	13	176	0.0002	ns	2.08	1.52
Clostridiales Family XI. Incertae Sedis	6	101	0.0467	ns	2.25	1.99
Corynebacteriaceae	57	632	3.0345	ns	1.58	1.24
Flavobacteriaceae	27	629	6.4096	<0.05	2.64	1.93
Lachnospiraceae	138	1508	9.3097	<0.01	2.46	1.88
Lactobacillaceae	11	148	0.0000	ns	2.58	1.68
Pelagibacteraceae	8	258	5.0707	<0.05	1.58	1.83
Planococcaceae	5	32	1.8696	ns	2.62	2.31
Porphyromonadaceae	7	40	4.0121	< 0.05	1.3	1.68
Prevotellaceae	13	99	0.0010	ns	1.56	1.49
Pseudomonadaceae	24	797	18.3400	<0.0001	1.09	1.24
Rhodobacteraceae	10	355	8.3811	<0.01	2.64	1.59
Rhodospirillaceae	10	211	1.2334	ns	1.23	1.21
Rikenellaceae	7	46	2.7799	ns	1.91	1.71
Ruminococcaceae	57	616	3.7217	ns	2.79	2.13
Staphylococcaceae	14	323	2.9319	ns	1.67	1.41
Streptococcaceae	76	209	186.8096	<0.0001	3.18	2.28
unclassified	38	618	0.5811	ns	2.13	1.89
Veillonellaceae	10	112	0.2855	ns	2.57	2.25
						
*More abundant in P. lutea*						
Comamonadaceae	101	903	20.4045	<0.0001	4.59	3.39
Desulfobacteraceae	6	88	0.0161	ns	1.28	1.09
Enterobacteriaceae	104	801	36.6887	<0.0001	2.66	2.08
Moraxellaceae	5	163	3.0366	ns	2.16	1.97
Propionibacteriaceae	6	65	0.1546	ns	1.57	1.09
Rikenellaceae II	32	332	2.5767	ns	1.53	1.27

Abbreviations: ANOVA, analysis of variance; NS, not significant; OTU, operational taxonomic unit.

**Table 5:** Over-/under-representation of bacterial families of OTUs differentially abundant between health states of *P. duerdeni* and *P. lutea*, and congregated fold-change differences between healthy (HH) and diseased (DD) specimens (only families that were represented by at least five bacterial taxa were considered)

*Bacterial family*	*OTU count ANOVA (total 629)*	*OTU count PhyloChip (total 14 213)*	*Chi-square*	P-*value*	*Mean fold-change difference between HH vs DD P. duedeni*	*Mean fold-change difference between HH vs DD P. lutea*
*More abundant in HH*						
Aquabacteriaceae	12	134	0.1030	ns	1.80	2.60
Bacillaceae	9	211	0.3936	ns	1.15	1.21
Burkholderiaceae	7	332	0.0000	ns	1.16	2.19
Comamonadaceae	6	903	18.3660	<0.0001	1.69	2.89
Enterobacteriaceae	20	801	6.4893	<0.01	1.41	2.07
Moraxellaceae	10	163	0.6779	ns	2.35	2.66
Streptococcaceae	23	209	17.3175	<0.0001	2.39	1.91
Xanthomonadaceae	7	120	0.2089	ns	2.48	3.29
						
*More abundant in DD*						
Alteromonadaceae	5	95	0.0171	ns	1.35	1.61
Clostridiaceae	5	176	0.6491	ns	1.78	6.41
Colwelliaceae	6	20	18.3660	<0.0001	3.94	4.08
Corynebacteriaceae	17	632	3.9726	<0.05	1.13	2.17
Flavobacteriaceae	27	629	0.0035	ns	3.18	2.97
Lachnospiraceae	35	1508	15.9206	<0.0001	1.22	1.73
Oceanospirillaceae	9	264	23.8756	<0.0001	5.35	7.84
Pelagibacteraceae	8	258	0.7250	ns	2.55	2.33
Pseudomonadaceae	57	797	12.6293	<0.001	3.12	4.66
Rhizobiaceae	11	97	8.0640	<0.01	3.58	1.93
Rhodobacteraceae	178	355	1150.8208	<0.0001	5.28	7.11
Rhodospirillaceae	9	36	0.0054	ns	2.39	2.79
Rikenellaceae II	7	146	3.3470	ns	1.83	1.55
Ruminococcaceae	11	616	9.3196	<0.01	1.15	1.01
Sphingomonadaceae	5	142	0.0901	ns	1.75	1.30
unclassified	25	618	0.1224	ns	1.73	1.48
Vibrionaceae	12	310	4.8116	<0.05	5.46	4.38

Abbreviations: ANOVA, analysis of variance; NS, not significant; OTU, operational taxonomic unit.
