# Supplementary figures and images for: Bacterial profiling of White Plague Disease in a comparative coral species framework
Source: ISME J. 2013 Aug 8;8(1):31–9. doi: 10.1038/ismej.2013.127 (PMC3869008; doi:10.1038/ismej.2013.127)

**A**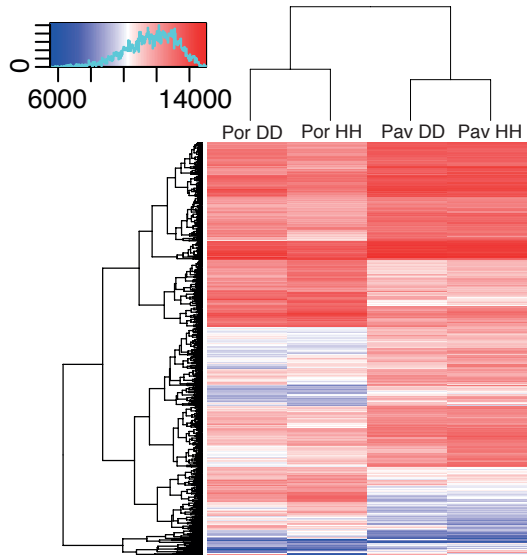**B**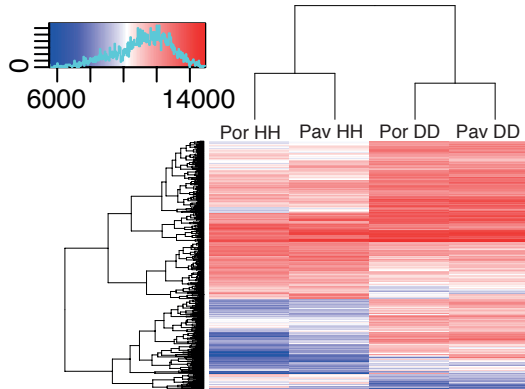

Supplement: Supplementary Figure [file ismej2013127x1.pdf]
