# Supplementary material for: Bacterial profiling of White Plague Disease in a comparative coral species framework
Source: ISME J. 2013 Aug 8;8(1):31–9. doi: 10.1038/ismej.2013.127 (PMC3869008; doi:10.1038/ismej.2013.127)
Supplement: Supplementary Legend [file ismej2013127x5.doc]

**Supplementary Information**

**Figure S1:** Heatmap diagram displaying clustered normalized HybScores of significantly differentially abundant OTUs (A) between species, and (B) between conditions. Color key and histogram are displayed on top left corner of heatmaps. HH: healthy, DD: diseased, Pav: *Pavona duerdeni*, Por: *Porites lutea*.

**Table S1**: HybScores for all present OTUs (n = 29,103) of PhyloChip^TM^ microarrays.

**Table S2**: Presence/Absence of 29,103 OTUs over all samples on PhyloChip^TM^ microarrays (0 = absent, 1 = present).

**Table S3**: Two-way ANOVA on 14,213 OTUs that were detected present in healthy and diseased specimens of the corals *P. duerdeni* and *P. lutea*.
